# Limited output transcranial electrical stimulation 2023 (LOTES-2023): Updates on engineering principles, regulatory statutes, and industry standards for wellness, over-the-counter, or prescription devices with low risk

**DOI:** 10.1016/j.brs.2023.05.008

**Published:** 2023-05-16

**Authors:** Marom Bikson, Ana Ganho-Ávila, Abhishek Datta, Bernadette Gillick, Morten Goertz Joensson, Sungjin Kim, Jinuk Kim, Adam Kirton, Kiwon Lee, Timothy Marjenin, Balder Onarheim, Erik M. Rehn, Alexander T. Sack, Gozde Unal

**Affiliations:** a Department of Biomedical Engineering, The City College of New York, New York, NY, United States; b Center for Research in Neuropsychology and Cognitive Behavioral Intervention–CINEICC, Faculty of Psychology and Educational Sciences, University of Coimbra, Coimbra, Portugal; c Research and Development, Soterix Medical Inc., Woodbridge, NJ, United States; d Department of Pediatrics, University of Wisconsin School of Medicine and Public Health, Madison, WI, USA; e Research and Development, PlatoScience ApS, Copenhagen, Denmark; f Ybrain Research Institute, Seongnam-si, Gyeonggi-do, South Korea; g Departments of Pediatrics and Clinical Neurosciences, Cumming School of Medicine, University of Calgary, Calgary, AB, Canada; h Regulatory Affairs, MCRA, Washington, DC, United States; i Research and Development, Flow Neuroscience, Malmo, Skane Lan, Sweden; j Department of Cognitive Neuroscience, Faculty of Psychology and Neuroscience, Maastricht University, Maastricht, the Netherlands

## Abstract

The objective and scope of this Limited Output Transcranial Electrical Stimulation 2023 (LOTES-2023) guidance is to update the previous LOTES-2017 guidance. These documents should therefore be considered together. The LOTES provides a clearly articulated and transparent framework for the design of devices providing limited output (specified low-intensity range) transcranial electrical stimulation for a variety of intended uses. These guidelines can inform trial design and regulatory decisions, but most directly inform manufacturer activities - and hence were presented in LOTES-2017 as “Voluntary industry standard for compliance controlled limited output tES devices”. In LOTES-2023 we emphasize that these standards are largely aligned across international standards and national regulations (including those in USA, EU, and South Korea), and so might be better understood as “Industry standards for compliance controlled limited output tES devices”. LOTES-2023 is therefore updated to reflect a consensus among emerging international standards, as well as best available scientific evidence. “Warnings” and “Precautions” are updated to align with current biomedical evidence and applications. LOTES standards applied to a constrained device dose range, but within this dose range and for different use-cases, manufacturers are responsible to conduct device-specific risk management.

## 1. Summary of updates from LOTES-2017 to LOTES-2023

The scope of this LOTES-2023 document is only an extension and update to the LOTES-2017 guidance. The sections updated and added are as follows:

1. The “Quality Systems” Part 1 sub-section in LOTES-2017 is replaced by the new LOTES-2023 section “2.1 Quality Systems, LOTES-2023 update.”
2. The LOTES-2017 Part 1 sub-section “Regulatory definitions and general standards” is replaced in LOTES-2023 by the section “2.2 Regulatory definitions and general standards, FDA-LOTES 2023 update.”
3. The LOTES-2017 Part 1 sub-section “FDA regulatory class and degree of controls” is replaced with “2.3 FDA regulatory class and degree of controls- LOTES-2023 update” including updates on CES and ECT classification.
4. We note the FDA draft guidance summarized in the LOTES-2017 section “FDA guidance on limited output specifications” has been withdrawn by the FDA, but in the absence of a revised document this remains the ‘last word’ from the FDA on the subject and so remains a reference for LOTES-2023. The LOTES-2017 Part 3 sub-section “FDA guidance on limited output specifications” is replaced in LOTES-2023 as “3.1 FDA guidance on limited output specifications, LOTES-2023 update.”
5. The “An approach to establishing Special Controls and Limited Output Guidelines for tES devices” section in Part 3 of LOTES-2017 is replaced with the new LOTES-2023 section “3.2 An approach to establishing Special Controls and Limited Output Guidelines for tES devices, US Guidance, LOTES-2023 update”. We thus emphasize this section addresses the FDA approach.
6. A new section “4.1 EU Guidance including Wellness” is added in LOTES-2023.
7. A new section “4.1 South Korea Medical Device Guidance” is added in LOTES-2023.
8. Warnings and Precaution are updated from LOTES-2017 to LOTES-2023 Section 5. This section includes a preamble (5.2, 5.3) explaining the updated general approach to Warnings and Precautions. The LOTES-2023 “5.2.1 Warning (LOTES-2023 update)” and “5.2.2 Precaution (LOTES-2023 update)” replace the respective LOTES-2017 Part 3 subsections “Warnings” and “Precaution”. These are followed by new sections regarding use in Pediatric (5.3), Pregnant (5.4, 5.4.1), and breastfeeding (5.5) populations which include justification for updated warnings and precautions.

The LOTES-2023 is the work product of only those authors listed, which differs in part from the LOTES-2017 contributors. The updated LOTES standard in totality is understood to include the prior LOTES-2017 document with the indicated enhancements in this LOTES-2023 publication, either updated sections or new sections. LOTES-2023 includes updates on minimal labeling regarding Precautions and Warning, while responsible labeling regarding efficacy remains as described in LOTES-2017. Providing technical guidance for the design, manufacture, and distribution of all limited-output tES devices, LOTES is distinct (but complimentary to) consensus-documents directed to device end-users for specific technologies and use-cases.

### 1.2. Scope of devices covered by LOTES 2017/2023

The scope of devices covered by LOTES-2023 remains unchanged from LOTES-2017. Output specifications as re-stated below. However, in order to meet LOTES standards, a device should satisfy the entirety of requirements as initially described in all relevant sections of LOTES-2017 (including on quality, safety, and labeling) and those updates in LOTES-2023.

- A maximum charge per phase that does not exceed Q, where Q = 20 + (28) (t) mC (and where t is the phase duration expressed in ms and measured at 50% of the phase amplitude);
- A maximum average current that does not exceed 10 mA;
- A maximum primary (depolarizing) phase duration that does not exceed 500 ms except as specified in vii;
- An average DC current that does not exceed 100 μA when no pulses are being applied, except as specified in vii, and does not exceed 100 μA when the device is inactive or when the device fails;
- A maximum current density that does not exceed an RMS of 2 mA/cm^2^ of electrode conductive surface area; and
- A maximum average power density that does not exceed 0.25 W/cm^2^ of electrode conductive surface area;
- For devices using direct current or continuous sustained current passage greater than 1 s, or square wave, or rectified or bias sinusoidal, or pulses with >25% duty cycle including all phases, if the maximum average current does not exceed 4 mA (average absolute value) then criteria (i), (iii), and (iv) are waived;
- A maximum peak output current that does not exceed 30 mA;
- A maximum current density that does not exceed 2 mA/cm^2^ of electrolyte-skin contact area;
- A maximum time per session that does not exceed 60 min;
- A maximum total charge per session that does not exceed 6000 mC;
- Electrode labeling: conditions for disposal or re-use specified.
- Skin tolerability testing conducted under electrode labeling conditions;
- Electrode testing consistent with draft guidance document Class II Special Controls Guidance Document: Cutaneous Electrode [1] or sufficient component-material documentation or usability testing;
- Electrolyte testing consistent with draft guidance document Class II Special Controls Guidance Document: Electroconductive Media [2] or sufficient component-material documentation or usability testing;
- Battery supply is exclusively used during stimulation. Should batteries be re-charged within the device, the device cannot stimulate during connection with an AC source (charging). During connection to an AC source, patient leakage current, including at the device outputs, should be at an acceptable level. In such cases, measurements of patient leakage current under both normal and single fault conditions should be recorded based on the FDA-recognized standard, IEC 60601e1, “Medical Electrical Equipment e Part 1: General Requirements for Safety” or an equivalent method to show that the measured levels of patient leakage current are acceptable;
- Any electronic equipment physically connected to the device, for example for the purpose of data collection or triggering, is considered part of the device for the purpose of these special controls;
- At least one electrode must be positioned on the head; if one or more electrodes are positioned below the neck, a risk analysis is required and waveform limits (i to xii above) should be further limited if necessary as determined by the outcome of this analysis.

## 2. Quality systems, LOTES-2023 update

This section replaces the sub-section “Quality systems” in Part 1 of LOTES-2017.

As part of effective risk management processes, it is necessary to ensure that device design and testing are conducted in an orderly way, and that manufactured devices uniformly meet the requirements of the design. Best practices here, also known as current good manufacturing practices (CGMP), are similar to the principles of quality assurance used in many industries (for example, the ISO 9001 standards). For medical devices, these principles are codified by FDA in the Quality Systems Regulation (QSR, 21 CFR part 820) and internationally by the ISO 13485 standard. It is of note that these regulations apply to a range of devices, and therefore rather than explicitly defining how a manufacturer must produce a specific device, they provide a template that manufacturers must follow to ensure orderly design, development, production, installation, and delivery. In practice, because CGMP regulations encompass a wide array of devices, it remains partially up to a manufacturer to establish a quality system and design process within these templates (FDA’s 21 CFR part 820 and/or ISO 13485), as well as establishing device requirements, that are sufficient to ensure safety and effectiveness.

In February 2022, FDA proposed to amend the requirements of the quality system regulation [3] to “align more closely with the international consensus standard for devices” through “incorporating by reference an international standard specific for device quality management systems set by the International Organization for Standardization (ISO), the 2016 edition of ISO 13485 (ISO 13485).” There are additional proposed requirements “to align with existing requirements in the Federal Food, Drug, and Cosmetic Act (FD&C Act) and its implementing regulations and make conforming edits to the Code of Federal Regulations (CFR) to clarify the device CGMP requirements for combination products.” Until finalized, FDA will continue to follow the existing quality system regulation.

### 2.1. Regulatory definitions and general standards, LOTES-2023 update

This section replaces the sub-section “Regulatory definitions and general standards” in Part 1 of LOTES-2017.

The FDA and comparable (trans)national agencies do not regulate the practice of medicine or general commerce. Rather, they prescribe quality and safety standards and regulations for the development and production of drugs and devices. As it relates to specific issues and questions fostered by limited output tES, it becomes important to clarify the definition of a “medical device”. Regulatory bodies and agencies of different countries have adopted varied positions and standards in defining a medical device. The United States has implemented a sweeping approach, which has served as a model for many international standards and codes. According to the FDA a medical device is:

“An instrument, apparatus, implement, machine, contrivance, implant, in vitro reagent, or other similar or related article, including a component part, or accessory which is:

- recognized in the official National Formulary, or the United States Pharmacopoeia, or any supplement to them,
- intended for use in the diagnosis of disease or other conditions, or in the cure, mitigation, treatment, or prevention of disease, in man or other animals, or
- intended to affect the structure or any function of the body of man or other animals, and which does not achieve any of its primary intended purposes through chemical action within or on the body of man or other animals and which is not dependent upon being metabolized for the achievement of any of its primary intended purposes.”

All medical devices are defined by FDA via classification regulations that are listed in the Code of Federal Regulations (CFR) by medical specialty. Each regulation briefly describes the device and its intended use, the classification, and any additional details that are applicable such as special controls. FDA has established three different classifications for medical devices, designated as Class I, Class II, and Class III depending on the risks imposed by device use and whether certain controls can be developed that will provide a reasonable assurance of safety and effectiveness. For example, dental floss, toothbrushes, and bandages are Class I medical devices. Non-invasive blood pressure monitors, condoms, and over-the-counter (OTC) transcutaneous electrical nerve stimulation (TENS) devices for treatment of pain or for aesthetic purposes represent examples of Class II medical devices. Heart replacement valves or deep-brain stimulation systems are examples of Class III medical devices. Based on the FDA definition of a medical device, and recognizing the spectrum of devices regulated, it is therefore the conservative position of this guidance document that tES technologies (i.e., tDCS, tACS, tPCS, etc.), whether indicated and/or employed for medical treatments, diagnostic purposes, wellness aids, recreational or entertainment devices, or any other purposes, may be classified as a medical device per the FDA - although others have argued to the contrary [4]. Importantly, jurisdiction does not equate with targeted regulation or enforcement – for example, non-medical treadmills or heaters are intended (and marketed) to “affect the body” but are in practice not controlled by the FDA as medical devices. Regardless, whether the FDA (or a comparable national agency) does not have jurisdiction or elects to not regulate, the LOTES guidance provides a necessary guidance for manufacturers (notably Part 3 in LOTES-2017 with the relevant updates section in LOTES-2023 including sections 3.1, 3.2, 4.1, 4.1, 5.2.1, 5.2.2).

The initial FDA classification regulations were created during a number of proceedings in the late 1970s and 1980s, during which devices already on the market at the time the Medical Device Amendments were enacted in 1976 was categorized. The FDA continues to create new classification regulations. Ongoing approvals and new classifications are developed in relation to the review of certain pre-market submissions that include de novo requests, PMAs, and HDEs. FDA does not preemptively create regulations for new devices that it may only become aware exist, and instead relies on a manufacturer’s submission – and a favorable outcome on that submission – in order to create the new regulation.

The FDA process of reacting to an industry proposal has implications for interpreting the absence of device/indication specific regulations. On the one hand, absence of a regulation defining a certain type of device and intended use, does not mean that FDA does not, or would not, regulate the device. Rather, it simply means there has been no submission (application from industry) on which they have been able to take favorable action. Regulations can also be changed once they have been created. However, this involves regulatory rulemaking, and is typically a long process. On the other hand, the absence of regulation also does not necessarily imply the FDA has made a negative determination regarding a specific device and indication. And even in cases when the FDA rejects a submission, this may not be (solely) based on insufficient evidence for benefit versus risk, but rather protocol details such as the outcome measure selected, what *average* changes are considered clinical meaningful, statistics, suitability of control arm etc. Similarly, to the extent the FDA may not regulate all wellness claims, absence of FDA approval (and a FDA classification) is not indicative of the FDA’s opinion.

Classification regulations are associated with product codes which are a way to subcategorize devices within a specific regulation (in addition to having post-market uses). One regulation may have more than one product code assigned to it; however, a product code will only apply to one regulation. In some cases, such as PMAs and HDEs, the regulation is not defined at the time of approval (unlike de novo), and product codes are used somewhat as a surrogate, until such time as a regulation can be created.

Closely related to the FDA classification of medical devices is application of the provisions of General and Special Controls. The basic provisions of the 1976 Medical Device Amendments to the Food, Drug, and Cosmetic Act provided the FDA with the means and authority to regulate devices in order to ensure their safety and effectiveness. Unless specifically exempted from regulation, all devices (Class I, II, and III) are subject to General Controls. Class I devices are only subject to General Controls, which include provisions that relate to misbranding, device registration and listing, premarket notification, banned devices, notification and repair, replacement and refund, records and reports, restricted devices, and Good Manufacturing Practices. Class II devices are subject to both General and Special Controls; and Class III devices are subject to General Controls and Premarket Approval (PMA). Special Controls are regulatory requirements imposed on Class II devices for which General Controls are insufficient, but for cases where there exists sufficient technical and clinical knowledge to confidently propose additional controls that will provide a reasonable assurance of safety and effectiveness. These Special Controls are typically device-specific and include upholding performance standards, premarket data requirements, special labeling requirements, and other guidelines. In the past, these might have been described in what was referred to as a “Special Controls Guidance Document” for a particular device, if they were defined at all – many Class II classification regulations do not identify specific special controls. More recently, these are established as part of the review of a de novo submission, and will be listed directly in the classification regulation. The PMA process is reserved for devices that are designated such that General and Special Controls cannot sufficiently provide a reasonable assurance of safety and effectiveness. This category of devices is subject to the most stringent of all FDA regulations.

In most countries, the intended use of a tES device, whether to treat a disease or to provide neuromodulation for consumer-oriented purposes, will determine if the device is regulated as a medical device or not. For example, a tES device indicated to treat depression will be regulated as a medical device in the majority of countries. However, depending on the country and application, the regulation of a tES device as medical or non-medical may vary; for example, tES device indicated to ‘enhance memory’. Even when a tES device is considered to fall outside a country’s medical device regulations, this does not imply, that such a (consumer-oriented) tES devices is not regulated by other safety standards (such as electrical and mechanical safety guidelines, or regulations regarding fair marketing of consumer products). A notable recent exception to exclusion of non-medical use tES devices from medical device regulations is the EU, where transcranial stimulation devices need to comply with medical device regulations regardless of whether or not they have a medical purpose (see section 4.1 for a more thorough discussion).

The International Electrotechnical Commission (IEC) has established globally accepted guidelines to ensure the manufacture of safe electrical products. Medical devices are specifically subject to the IEC 60601 family of standards, most importantly the general safety controls in IEC 60601–1. This general standard is well harmonized between the international (IEC) and United States’ (Underwriters’ Laboratories-UL) versions, and compliance with the IEC standards is often required to receive medical device approval within the EU. Collateral standards in the 60601 family cover topics such as equipment’s alarm systems, equipment for home health care environment, electromagnetic immunity and emissions of the equipment, equipment’s performance and usability, programmable electrical medical systems equipment, nerve and muscle stimulators, electroconvulsive therapy equipment [5]. Some types of devices are also subject to specific standards. However, note that IEC 60601–2-10 explicitly excludes tES devices from its scope. Similar to IEC, the International Organization for Standardization (ISO) establishes standards but which are focused on general market relevant ones to support innovation, facilitate trade, and enable standardization in all industrial fields. The main standards that all medical devices are subject to, include ISO 14971 (risk management) and ISO 10993 (biocompatibility). Additionally, manufacturers are subject to region-specific guidelines such as the guidance documents provided by the FDA that cover software, cybersecurity, human factors etc.

Irrespective of definition and jurisdiction by agencies that govern medical devices, jurisdiction does not imply or mandate targeted regulations i.e., a product might be considered a medical device and fall under the jurisdiction of a regulatory agency such as FDA according to the statutory definition, but the agency may choose not to explicitly examine the product. Further, there is recognition that even when devices are not strictly defined as medical the importance of risk management remains paramount. Thus, notwithstanding these differences, across countries, any tES device, regardless of indication for use, would be generally regulated by rigorous design, production, and often distribution standards such as CE mark in Europe. As well, further legal restrictions may apply. For example, as previously noted, in the United States, marketing claims for tES devices fall under the purview of either the FDA, and/or the FTC, which serves to protect consumers against false labeling and advertisements. Thus, in most countries, including the United States, debating “if” tES devices should be regulated is a moot point, since regulations under international and regional laws already exist that specify engineering standards and rules for distribution. Part 3 in LOTES-2017 with the relevant updates section in LOTES-2023 including sections 3.1, 3.2, 4.1, 4.2, 5.2.1, 5.2.2 thus provides guidance on best practices that align with existing rules.

### 2.2. FDA regulatory class and degree of controls, LOTES-2023 update

This section replaces LOTES-2017 Part 1 sub-section “FDA regulatory class and degree of controls”.

For any tES device intended for a purpose of providing therapeutic intervention against a disease or medical condition (i.e., a medical label), medical device regulation by the FDA is further subject to the particulars of regulatory class. There are several possible regulatory paths within the FDA by which a product can be approved for market. First, a device or product can be exempt from a clearance path, which is often referred to as a 510(k) exemption. Nearly all Class I Medical Devices are exempt from regulatory clearance paths, in the sense that formal submissions to the FDA for marketing are not required, assuming that a manufacturer meets relevant regulations and international guidelines (e.g., IEC-60601).

Most non-invasive electrostimulation devices that are in some way similar to tES in function and application, such as TENS, powered muscle stimulation (PMS), and iontophoresis devices are Class II medical devices and, as such, are regulated by a Premarket Notification 510(k) process. These devices may be cleared for prescription or OTC use. In the past, most electrostimulation products cleared through the 510(k) process have been designated as prescription-use devices, for example, TENS devices that were intended to treat pain. However, with increased confidence in the safety of electrostimulation technologies and devices gained over the past four decades, and the appropriate evidence, many such products have now been cleared for OTC use. Thus, TENS devices, whether used to treat pain or for aesthetic purposes, as well as PMS devices for muscle conditioning, can now be marketed for OTC use and purchased at many pharmacies and/or online once they have been cleared by FDA.

Under the 510(k) path, the FDA can deem a new device intended to be marketed to be substantially equivalent to a “predicate” device that is already on the market for a specific indication. If cleared, the FDA will assign the product code of the predicate device for a specific intended use. If the device either has a new intended use compared to the predicate or technological differences compared to the predicate raise different questions of safety or effectiveness, a device manufacturer may seek marketing authorization through the de novo classification process, which results in the creation of a new classification regulation. For example, the *Cefaly* device, a TENS unit indicated to relieve pain associated with headaches via the use of low-level electrical current delivered through electrodes placed on the head, recently received marketing authorization from the FDA through this de novo process (21 CFR 882.5891, product code PCC). The *Cefaly* device was initially available for prescription use only. However, in 2020 Cefaly also received clearance for OTC use of their device.

In 2008, the FDA gave marketing authorization to the *Neuronetics (Neurostar*) device, which uses repetitive transcranial magnetic stimulation (rTMS), via the de novo pathway, under a new regulation 21 CFR 882.5805 (product code OBP). *Brainsway* (Deep TMS) rTMS followed in 2013. Other rTMS products have since been cleared under the 510(k) process. The use of high-intensity repetitive pulsing by these devices is associated with a low risk of seizures [6]. It is noteworthy that a broad range of energy output devices have been classified as Class II and/or cleared through the 510(k) process.

Since the prior LOTES publication, the classifications for both electroconvulsive therapy (ECT) and cranial electrotherapy stimulators (CES) have been finalized. These were remaining two devices for which FDA had yet to formally finalize classification from a group of devices in a similar regulatory situation. In both cases, the classifications were split, with some intended uses designated Class II and others as Class III on the basis of whether sufficient data on safety and effectiveness existed at the time for those uses. ECT devices provide current across the head with 500–900 mA intensities, which fall outside the scope of this LOTES document. CES devices have traditionally used limited output current, and so it may seem odd for part of the final classification of CES to be Class III (as is the case when intended to treat depression). However, in this particular instance the claimed issue was a lack of sufficient evidence from which FDA could develop special controls. What is important to recognize is that CES for depression could be reclassified in the future in the event that sufficient evidence is collected to support the development of special controls, and there is regulatory action to change the classification.

In 2010, the FDA published Class II Special Controls Draft Guidance Documents for Industry and FDA staff, which if put into effect, would make certain OTC TENS and PMS devices with specific medical or aesthetic labels exempt from 510(k) [7,8]. This draft appears to have been rescinded, and there is currently no successor draft. The premise of these documents was to establish Special Controls with Limited Output guidelines for several types of OTC electrostimulation products with medical indications that are designated as Class II devices. For example, TENS with limited outputs for aesthetic purposes, and PMS with limited outputs for muscle conditioning, are both intended for OTC consumer use and were proposed to be exempt from 510(k). It is critical to note that these devices would have remained Class II devices and still required to verifiably comply with certain General and Special Controls, as well as international safety standards. The draft guidance documents were important because they established defined parameters with which to deem and ensure the safety of electrostimulation products of various types (regardless of indication for use), as based upon the bulk of evidence available to date. Therefore, while the draft guidance is no longer available, the industry guidance we propose (Part 3 in LOTES-2017 with the relevant updates section in LOTES-2023 including sections 3.1, 3.2, 4.1, 4.2, 5.2.1, 5.2.2) for limited output tES devices with medical (prescription or OTC) or wellness labeling remains harmonized with this draft FDA guidance.

In Part 2 of LOTES-2017 (*Engineering analysis of electrostimulation output parameters*) we described the range of waveform parameters produced by FDA cleared (OTC) devices, which constrains the scope of devices included in this industry guidance. Table 1 of LOTES-2017 depicted different electrostimulation devices for which FDA product codes presently exist, including five product codes for OTC electrostimulation devices with limited output. These devices encompass, and in many cases exceed, output characteristics of state-of-the-art tES methods that are currently being used. There is also overlap in use; for example, iontophoresis devices have been used off-label in numerous tDCS clinical trials (with IRB non-significant risk designation). Table 1 of LOTES-2017 also indicates the Medical Device Class for each product code, the regulatory path for obtaining clearance, whether a device is indicated for OTC use, and whether a device is intended for use on the head. The specific output characteristics of these devices were discussed in LOTES-2017.

## 3. FDA guidance on limited output specifications, LOTES-2023 update

This section replaces the LOTES-2017 Part 3 sub-section “FDA guidance on limited output guidance.”

As previously stated, the 1976 Medical Device Amendment afforded the FDA authority to establish General and Special Controls for regulating devices. The FDA established draft guidance documents specifying Class II Special Controls and Limited Output guidelines for certain types of OTC TENS and PMS devices, after nearly 40 years of well-tolerated use of such devices [7,8]. TENS devices for the treatment of pain, and for aesthetic purposes, and PMS devices for muscle stimulation can be purchased OTC, but at present these devices must still obtain clearance through the 510(k) process. If and when any future documents are put into effect and incorporated to the FDA regulatory practices, such devices will no longer be required to gain clearance through the 510(k) process prior to marketing the device in the United States. They will, however, continue to be subject to both General and Special Controls, and must adhere to international safety guidelines such as IEC-60601. According to the previously-released FDA Draft Guidance documents [7,8] Limited Output specifications are met when: the device utilizes a stimulus generator that delivers, into a resistive load the worst case of either 500 Ohm or the typical load expected during normal conditions of use, the following:

- A maximum charge per phase that does not exceed Q, where Q = 20 + (28) (t) mC (and where t is the phase duration expressed in ms and measured at 50% of the phase amplitude);
- A maximum average current that does not exceed 10 mA (average absolute value);
- A maximum primary (depolarizing) phase duration that does not exceed 500 ms;
- An average DC current that does not exceed 100 μA when no pulses are being applied, or if the device fails;
- A maximum current density that does not exceed a root mean square (RMS) value of 2 mA/cm^2^ of electrode conductive surface area; and
- A maximum average power density that does not exceed 0.25 W/cm^2^ of electrode conductive surface area.

In order to assure device safety, the FDA had recommended additional guidelines; these included the following:

- The output of the stimulus generator should be controlled by appropriately marked knobs, dials, switches, indicators, etc., and these controls should modulate output intensity in a smooth, incremental, and predictable manner.
- The stimulus generator should not become unsafe if the output is switched on with open-circuited or short-circuited electrodes. Battery supply voltage fluctuations of ±10% should not affect the stimulus generator output amplitude, pulse duration, or pulse repetition frequency (rate) by more than ±10%.
- The stimulus generator should be limited to use with a battery power source, should be isolated from earth ground, and should not be capable of use with an AC power source or use while connected to a battery charger.
- All skin-contacting materials should be biocompatible for their intended use. To determine the applicable device category and tests, it is recommended to consult ANSI/AAMI/ISO 10993e1:2003, “Biological evaluation of medical devices – Part 1: Evaluation and testing.” This FDA-recognized standard recommends evaluation and testing of medical devices based upon the duration and type of contact. For cutaneous electrodes with a limited contact duration (e. g., less than 24 h), the standard recommends the following tests to establish material safety: dermal irritation, sensitization, and cytotoxicity.

These documents support the safe use of several products that use electrodes placed upon the head and face for a variety of differing intentions and purposes (e.g., TENS devices for cosmetic or aesthetic purposes). We posit that even in the absence of a current draft, the previous draft Special Controls establishing limited output guidelines are an appropriate framework for regulating tES devices for lifestyle and general wellness use, and can also inform the regulation of prescription-or OTC tES devices to treat certain neurological or psychiatric conditions.

### 3.1. An approach to establishing Special Controls and Limited Output Guidelines for tES devices, LOTES-2023 update

This section replaces the LOTES-2017 Part 3 sub-section “An approach to establishing Special Controls and Limited Output Guidelines for tES devices.”

As elements of a classification regulation, special controls can be established in one of two ways. The less common approach would be for FDA to pursue formal rulemaking processes to update an existing regulation. The more common approach is currently to establish them as part of the review of a de novo application for a new device type. The decision letter for a de novo includes what is referred to as a risk mitigation table, which outlines what FDA believes are the clinical risks to health, and the high-level mitigation measures. Those mitigation measures are further defined within the special controls. Each special control maps directly to a risk mitigation measure. Among tES devices, there are several common risks and therefore, likely common special controls. For instance, “adverse tissue reaction” is typically cited for devices with patient-contacting components. The associated mitigation measure would be “biocompatibility evaluation,” and the special control would say “The patient-contacting components of the device must be demonstrated to be biocompatible.” Another risk might be “electrical, mechanical, or thermal hazards that may result in user discomfort or injury (e.g., electrical shock or burn).” There can be multiple mitigation measures for a given risk, and in this case those might be characterized as “non-clinical performance testing,” _“_electrical, mechanical, and thermal safety testing,” “electromagnetic compatibility (EMC) testing,” “software verification, verification, and hazard analysis,” and “labeling.” Each would have an associated special control; in the case of labeling, there may be device-specific warnings or other language. When developed recently, special controls will not generally cite specific consensus standards; instead, they will describe what must be demonstrated (e.g., biocompatibility, or electromagnetic compatibility). Existing special controls for tES devices are instructive as they provide an initial set of considerations that can inform the testing that may be necessary. Decision summaries for de novo submissions include additional information regarding how devices were evaluated that can be useful. The LOTES “Recommended industry standards for compliance controlled limited output tES devices” and LOTES quality and performance standards (Part 3 in LOTES-2017 with the relevant updates section in LOTES-2023 including sections 3.1, 3.2, 4.1, 4.2, 5.2.1, 5.2.2) are harmonized with this approach as applied by the FDA (and other regulatory agencies) to limited output electrical stimulation devices, while limited to least-burdensome and evidence-based requirements.

As discussed above, and elsewhere in the literature [9–11], tES has a substantive history of safe use when quality devices are aptly used in accordance with established protocols. Hence, both device design (e.g., waveform output, electrodes) and usability (e.g. headgear, accessory preparation) are important considerations for regulation. Based on existing FDA regulations and historical precedent(s), limited output tES intended for medical therapy would mostly likely be classified as a Class II device, and subjected to different clearance pathways dependent upon intended use and indications for use. A necessary distinction must be made for tES products that are marketed for intended use in diagnosing or treating a medical condition or disease. Such devices would require instructions and close monitoring by a physician. Therefore, any tES device marketed by a manufacturer to treat a medical condition (e.g., depression, anxiety) would only be cleared for prescription use, unless explicitly cleared for OTC use. Prior to clearance for marketing, devices intended for medical or diagnostic applications must meet the burden of proof to demonstrate efficacy through appropriate clinical trials when necessary.

On the other hand, direct-to-consumer (DTC) provision would be appropriate for limited output tES devices that are marketed to optimize certain cognitive abilities or to achieve certain wellness goals, such as stress reduction, and which are not intended for treatment of a medical condition. Restriction of output parameters as described herein (which are in accordance with the aforementioned draft Class II Special Controls guidance) would provide adequate safety assurances. Additionally, marketing strategies and claims made by tES manufactures of consumer-directed devices would be subject to United States FTC regulation (and/or procedures of regulatory bodies in other countries; see above).

Provided devices meet the criteria for limited output tES, existing standards would provide guidelines for device design and dissemination, as reflective of a history of safe use of similarly regulated low-intensity electrical stimulation devices. Importantly, recognition of existing laws illustrates the distinction between medical-grade technology and so-called do-it-yourself (“DIY”) brain stimulation devices. There is an active community of so-called DIY enthusiasts that are devoted to “at-home” construction of tES devices, sharing open-source plans for building tES devices. The ad hoc nature of the design (e.g., not following an exhaustive risk management protocol), manufacture (e.g., incomplete controls and documentation), usability (e.g., prone to misuse), testing (e.g., strict quality systems), marketing/distribution (including labeling), and monitoring (e.g., established protocols for documenting and responding to user reports) of DIY devices position them outside of the limited output tES guidelines and standards. Our guidance does not advocate restricting access, but rather providing consumers access to specifically defined and controlled quality devices.

We believe that available regulated paths for the responsible commercialization of limited output tES devices (with which LOTES-2023 is aligned) make controlled products available to users (with appropriate labeling as suggested by LOTES-2023), and additionally can prevent injuries that may be incurred through the use of adulterated tES products by the DIY community. It is important to emphasize that companies commercializing limited output tES devices, regardless of intended use, should adhere to Good Manufacturing Practices and all applicable international and regional standards. This industry guidance provides a framework for such compliance.

Clinical treatment (i.e. the practice of medicine) is also regulated by strict federal and state medical standards, and there is an ethical and legal imperative and obligation for health care providers to be well informed by current evidence demonstrating safety and effectiveness of therapeutic approaches employed, inclusive of neurotechnologies such as tES [12–14]. As for any on- or off-label interventions, for limited output tES devices these standards provide a framework for care. Standards for human trials are also well established. Based on ongoing practice (experience) with limited-output tES, these general standards are necessary and sufficient for patient protection (i.e., special standards that increase regulations specifically for limited-out tES are not productive). The unjustified obstruction of human trials has the consequence of undermining the generation of evidence for and against interventions. Undue regulation that prevents a physician from prescribing a therapy known to have evidence-based benefit in treating a particular patient diagnosis violates the probity of medicine, and undermines the value of regulatory action to insure sound care [15]. Moreover, failure to make available certified (e.g., medically/LOTES-2022 standards) tES devices to physicians/patients can also promote user pursuit of untested and unsafe devices.

As appropriate, clinicians may direct patients toward: 1) Direct-to-Consumer limited output tES devices which are otherwise available to healthy individuals and/or 2) Prescription/OTC tES devices marketed for the given indication or for off-label use. Prescription devices may not necessarily need to be held to restrictive limited outputs, but can be as governed by this guidance. As emphasized above, all tES devices, including those with limited output, are under the jurisdiction of FDA or other international regulatory agencies and consequently may be considered medical devices, and are therefore subject to extensive design and production standards. Thus, it will be increasingly important to define which tES devices are limited output and compliance controlled.

## 4. LOTES-2023 update on regulations: EU

In May 2021 a new Medical Device Regulation (MDR) 2017/745 came into full force within the European Union (EU). MDR replaces the Medical Device Directive (MDD) 93/42/EEC, making it mandatory law (not just a set of recommendations) for EU’s member states and manufacturers looking to place products in the EU. The new regulation modernizes the legislation surrounding medical devices, to, for instance, better encompass pure software devices (e.g., smartphone applications), and tightens the requirement around clinical evaluation and post-market surveillance to prove both safety and effectiveness for the intended use.

Importantly for tES device manufacturers, the MDR not only covers devices with a stated medical purpose but explicitly regulates tES devices which are similar to medical devices in terms of functioning and risks profile. Annex XVI of the MDR lists a set of device categories that, despite lacking an intended medical purpose (and so would generally not be medical devices) are still covered by the regulation. Notable categories are contact lenses and various devices for cosmetic surgery, but in the final Section 6 “Equipment intended for brain stimulation that apply electrical currents or magnetic or electromagnetic fields that penetrate the cranium to modify neuronal activity in the brain” is listed. This implies that all tES devices sold within the EU, regardless of whether or not the intended use is medical, fall under the MDR. Currently this has been understood by Notified Bodies to mean that Class II approval is needed for any tES device, but a recent EU draft act proposes that tES devices without an intended medical purpose should all be considered Class III [16]. It is the authors understanding that the proposed reclassification does not impact medical tES devices which can still be classified as IIa according to Rule 9 of the MDR Annex VIII. Regardless, all tES devices in the EU will need approval of a Notified Body to get the CE-certificate required to be marketed and sold within the EU.

We note LOTES-2017 (and by extension LOTES-2023) includes in its scope of Limited-output tES any devices that apply electrical stimulation across the head including both devices with nominal sub-cranial targets (eg. brain) or extra-cranial targets (eg. cranial or peripheral nerves). This is under various rationales notably the dose overlaps of such devices (LOTES-2017 section “Sub-vs extra-cranial targets). The MDR adopts the more traditional definition (“transcranial”; [17]) requiring “fields penetrate the cranium to modify neural activity in the brain.”

TES manufacturers need to comply with ISO 13485, including the new standard EN ISO 14971:2019 for risk management. These requirements along with the external approval of a NB, align well with the proposed industry standards outlined in the LOTES-2017 paper and reflected in this LOTES-2023 update. It indicates that the EU regulatory bodies fundamentally agree with the assessment made in the initial LOTES-2017; the procedural difference being the transition from largely self-regulation by manufacturers to oversight by a Notified Body.

Existing medical devices that were approved under the old MDD at the time of the formal release of the MDR May 26, 2021, are currently under a transition ‘grace period’ until May 26, 2024. These devices can be sold in compliance with the old MDD but are required to be in the process of an MDR upgrade leading up to the May 2024 deadline in accordance with MDR Article 120.

So far, the main challenge for MDR compliance for any tES device, regardless of intended purpose, has been that the common specifications for the groups of products without an intended medical purpose listed in Annex XVI have not yet been published. The Medical Device Coordination Group (MDCG) responsible for publishing the common specifications, published a draft document Ares (2022)271416 on 14/01/2022 for stakeholder hearing, and after a long process the final version of the common specifications C(2022)8626 was adopted on 01/12/2022.

The common specifications take a restrictive approach to “specific risk control measures” for some populations (e.g., pregnant women, persons less than 18 years old, or individuals with “sleep disorders”) requiring “specific evidence for safe use.” The consequence of this will be that (except for limited cases where the clinical disorder overlaps with the “specific risk control measures”) manufacturers will default to excluding these individuals (e.g., a depressed pregnant woman in the EU would be excluded from treatment) even in the absence of evidence for risk. In some cases, there may be limited evidence for safe and effective use in the very group being excluded. This conservative approach aligns with some other medical regulations as well as the original LOTES-2017. The LOTES-2023, however, modifies *minimal* “warnings” and “precautions” to be evidence based (5.2, 5.3); where a risk analysis that excludes special risk to a subpopulation is a basis to not automatically deny that subpopulation benefit, as opposed to the need to run special safety trials in each subpopulations, which for almost all devices/subpopulations will simply never happen. This distinction does not result in an inherent misalignment with MDR, except that individuals in the EU under these additional “blanket” restrictions would be limited in accessing devices/therapies (e.g., a cancer patient in the EU with a tumor cannot use a tES device indicated for pain management.)

Furthermore, an important takeaway from the common specifications is that they are also very specific on detailed instructions for manufacturers, for instance specifying that instructions of use “shall contain the internet address where the videos with instructions (…) can be found”. Here, the regulators are providing design directions, and thus potentially limiting novel developments in the field, such as other digital solutions not including internet addresses.

Additionally, for tES products without an intended medical purpose, a challenge is that the Class IIa classification has traditionally been used for devices with a clear medical purpose, which includes the requirement for a Clinical Evaluation Report (CER). A clinical evaluation report requires establishing evidence for safety, effectiveness and from these determining if the benefits of a given device outweighs the risks. It is currently unknown to the authors which requirements will be applied for a CER not aiming at medical application of tES as benefits can be hard to define rigorously and thus difficult to compare to definite risks.

The extension of EU medical-device regulations to all tES devices, ambiguity regarding the application of these regulations (including to on-going and long-standing practices in research), and opaqueness as to the process/rationale of the regulation development, has resulted in disruptions and has been protested [18–20]. A final logistical issue for compliance with the new MDR requirement is the delay in approval of Notified Bodies under the MDR, leading to a lack of organizations legally able to perform the required audits before the ultimate deadline for MDR compliance.

### 4.1. LOTES-2023 update on regulations: South Korea

South Korea, like other countries, also determines whether the intended use of tES devices is regulated as medical devices, depending on whether to treat diseases or provide neuromodulation for consumer-oriented purposes. In particular, tES devices indicated to treat depression or to improve cognitive abilities in patients with mild cognitive impairment or dementia are regulated as medical devices. In order to penetrate medical device market in South Korea, approval for medical device manufacturing business and items by the Ministry of Food and Drug Safety (MFDS) is required, and if used by doctors, approval of new health technology evaluation (HTA) by the National Evidence-based Healthcare Cooperation Agency (NECA) is also required. Medical device approval system of MFDS is similar to FDA and MDR standards. MFDS classifies medical devices into four classes according to their purpose of use and risk, and approval must be obtained for class 2 or higher. Since January 2022, cyber security-related approval for medical devices have also been executed. In South Korea, electrical stimulators are classified as class 2 or 3 medical instruments, and tES for depression treatment or cognitive improvement is classified as class 3 brain electrical stimulator for psychotherapy.

In South Korea, all tES manufacturers shall obtain Korean Good Manufacturing Practice (KGMP) same as ISO 13485:2016, including ISO 14971. With the introduction of the 5-year medical device renewal system, which took effect in July 2021, the approval must be renewed before the expiration of the validity period of the medical device item approval. Through this, medical device production/import performance and safety and effectiveness are regularly reviewed.

In December 2015, MFDS produced and distributed the approval guidelines for an electrical stimulator for brain and psychotherapy, which are medical device items of tES. The guidelines present safety and performance evaluation items as electrical stimulation devices and explain the specifications of evaluation items. Testing on electrical and mechanical safety and testing on electromagnetic safety shall comply with the common reference specifications for medical devices. For a test on the performance as an electrical stimulation device, the power and operation are evaluated in accordance with IEC 60601-2-10, and the power standard and control method are evaluated in accordance with the 510(k) FDA guidelines. In addition, guidelines for clinical trial are also presented.

In South Korea, tDCS, as a part of tES, was approved as medical devices with import approval in 2014 and manufacturing approval in 2016, with CE-MDD equivalence, for the purpose of improving the cognitive ability of depression patients to take antidepressants and rehabilitating finger movements. However, only limited use was allowed in the clinical use because HTA was not cleared by NECA. In year 2021, MFDS of South Korea approved the first tDCS (MINDD STIM+, Ybrain Inc., Korea) for at-home treatment of mild to moderate major depressive disorder under doctor’s prescription. The final approval decision of MFDS for the first indication of tES was made conservatively based on the inclusion and exclusion criterion of the premarket approval study design. The first premarket approval study proved 57.4% of remission rate in Beck Depression Index-II (BDI-II) in mild to moderate major depressive disorder patients after 6 weeks of at-home self-administration treatment. During the study period, no serious adverse event has been reported [21]. In addition to the first approval of the tES based medical device, there has been significant progress in actual medical practice using tES in South Korea. In June 2022, NECA, a regulatory body that evaluates new medical technologies and provides the list of proven medical practices of South Korea by law, decided to temporarily list the at-home tES as a medical practice for the treatment of mild to moderate major depressive disorder. Therefore, in South Korea, it has become possible to use tES as a medical device for the purpose of improving depression in patients with major depression disorders. A confirmatory clinical trial that evaluates the effectiveness and safety of cognitive function improvement using tES in mild dementia patients has been approved by MFDS in March 2019.

Table 1 shows the statistics of tDCS in South Korea under real-world conditions from June 2018 to February 2022. In approximately 45 months, 4866 users conducted 172,536 tDCS sessions. All use was conducted under the supervision of the physician and there were no serious adverse events. The intensity of stimulation was 1 mA–2 mA, 69.81% of the stimulation was 2 mA, and the stimulation time was 5–60 min, 66.21% for 30 min, and 27.79% for 20 min. Among the 4866 users, there were 141 children under the age of 10 and 786 adolescents under the age of 20. Also, there were 1377 women of childbearing age between the ages of 20 and 49, and none of them had any serious adverse events. MINDD STIM was developed and approved for at-home use by patients. 37,918 sessions were used by 3157 users in the hospital, while 134,648 sessions were used by 1921 users in the at-home use. The average number of stimulation sessions per user was 12 at the hospital and 70.1 at home.

## 5. Updates to warnings and precautions: General approach

Device/use specific labeling of ‘Warnings’ and ‘Precautions’ should follow best practice of risk management as prescribed in LOTES-2017. This includes identifying risks and establishing mitigation measures that are aligned with data on risk, to ensure risks are at an acceptable level and the known benefits outweigh these risks. ‘Warnings’ and ‘Precaution’ labeling may be dynamic and should be updated accordingly as additional data becomes available. The updated LOTES-2023 ‘Warnings’ and ‘Precaution’ are listed below and replace LOTES-2017 ‘Warnings’ and LOTES-2017 ‘Precautions’. However, (as stipulated in LOTES-2017): Labeling recommendations are not intended to represent all possible limitations or strict instructions for tES devices with limited output. Therefore, when developing adequate directions for use, it may be necessary to modify limitations (e.g., contraindications, warnings, precautions, adverse reactions), and other instructions that are appropriate for the specific device, depending both upon on its specific design (features, performance characteristics) and intended use, including based on results and conclusions drawn from usability studies.

The approach to recommend warnings and precautions is guided by the LOTES standard spanning 1) a wide class of limited output tES devices, 2) with a range of case uses, ranging from wellness to medical and 3) representing a baseline, but not absolute, recommended standard. For example, the label for a medical device will rationally require an adequate level of evidence for safety in all populations and use cases in the label, and therefore implicitly or explicitly (through warning and precautions) placing all other populations and use cases outside the label (i.e., off label).

Based on the approach stated above, LOTES-2023 considers reasonable evidence for risk (which can include empirical evidence in use or quantitative and specific theoretical considerations such as models) as necessary in order to recommend a baseline “precaution”. Absence of evidence is not sufficient for a precaution. Our standard for baseline “warning” is more relaxed including calling out susceptible populations even in the absence of evidence to risk - even so, our language is carefully not to imply evidence for risk but rather suggest vigilance or supervision. Notwithstanding this, the omission of a use case from the LOTES is not an endorsement of safety (certainly not for any feasible low intensify tES device over and dose/period of use). And the responsibility remains on the manufacturer to develop appropriate labels based on risk analysis as indicated in LOTES 2017 as well as based on the latest and evolving evidence base.

Unknowns about the mechanisms of limited output tES on the workings of the brain (e.g., human development across the lifespan) do not in themselves support a precaution based on the LOTES approach (as they may result in an argument-from-ignorance or slippery-slope logical fallacy). However, a more conservative standard may be applied to baseline warnings when considering susceptible populations. These warnings, however, need to be carefully phrased as to not suggest evidence for risk where none exists.

The use of limited output tES, whether self-directed or under prescription, should be guided by risk vs benefit analysis. Benefits are evidently use specific. Risks must always also be weighed against alternatives (eg. disease progression in the absence of any care including a harm of omission; side effect profile of treatment alternatives in vulnerable populations). This is similar to the requirements for clinical evaluation from the MDR. Under section 513 (a) of the Federal Food, Drug & Cosmetic Act (the “FD&C Act”), FDA determines whether PMA applications provide a “reasonable assurance of safety and effectiveness” by “weighing any probable benefit to health from the use of the device against any probable risk of injury or illness from such use,” among other factors-as based on scientific evidence. The FDA provides further guidance on its consideration for Benefit-Risk Determinations in multiple situations, although the most relevant here is the one that targets premarket applications such as PMAs, de novos, and HDEs [22].

Just as risks should be determined based on a scientific evidence base, so should ‘warnings’ and ‘precautions’ – avoiding both excessive or insufficient messaging. For wellness or neuroenhancement the benefits may be harder to delineate and/or more individual goal directed, but the risks – especially as they impact precautions and warnings – would *a priori* be the same. As LOTES is intended to provide general (not use specific) guidance, LOTES cannot make risk/benefit recommendations. None-the-less, the LOTES baseline precautions and warnings (along with supporting text) are based on and so should support evidence-based decisions. For this reason, ‘precautions’ declared in the absence of evidence can be considered disinformation in the specific context of the LOTES guidance - as noted about in other contexts such as medical label precautions may serve a different function.

It is important to contrast the scope of LOTES with safety consensus document prepared by and directed to clinician/scientists [6,23–26]. Such safety consensus documents are complementary to LOTES but are distinct in 1) limiting conclusions to sub-categories of technologies (e.g., just tDCS; [23]) and/or indications (e.g., vision modulation; [27]; neuroenhancement [28], addiction [29]) and/or use-cases (e.g., remote-supervised treatments [30]; use in MRI; [31]; COVID restrictions [32]); which in turn allows 2) more specificity in regard to best practices; 3) adopting document specific protocols on why/how standards are recommended; which are thus 3) directed to device end-users and laws/ethics governing end-users; 4) and may address further topics such as mechanisms. Methods [33–35], nomenclature [36], or data transparency [37,38]. Per its explicit scope, LOTES provides broad standards for the design, manufacture, and distribution of limited-output tES devices.

### 5.1. Updates to warnings and precautions: rationale of changes from LOTES-2017

Guided by the above principles, the LOTES-2023 removes or qualifies several Warnings and Precautions in LOTES-2017. Statements that are 1) sufficiently vague as to confound implementation; 2) lack evidence-base for risk (see above); and/or 3) contrast with existing use-cases are removed. For example, warnings/precautions regarding implants can be ambiguous regarding what types of devices qualify (e.g., dental implants), are not supported by evidence for risk, and contradict with use-cases of stimulation with intracranial implants [39–43] or pacemakers [44]. There is also evidence for the use of electroconvulsive therapy (ECT), which involves intensities above (~400x; [45,46]) those relevant for this limited-output guidance, with implants without injury [47–49].

We conclude it does not make sense to issue “blanket” warnings against use cases that are in fact investigated applications of limited output tES. For example, if there have been human trials (under non-significant risk designations) of limited output tES to boost an activity, moreover with positive outcomes, it seems not-evidence based to issue a “ban” against that same activity during tES. Precedent of a factor being an exclusion criterion for a trial or being a “warning” on a (FDA) device labeling, is not in itself evidence for risk. For example, whereas history of seizure, or cranial defect, or implants are often exclusion criteria, human trials of subjects with the combination of epilepsy (moreover deprived of medications), acute skull defects, and extensive implants have been conducted without adverse events. Finally, we note even theoretical risks should be based on quantitative and scientific considerations, rather than ad hoc rules (or logical fallacies). Taken together, we are concerned that the pattern of reiterating certain warnings (or contraindications) can create precedent that is then confused with actual evidence (a basis for) risk.

Warnings/precautions regarding use while operating machinery or during otherwise risky activities can also be vague or in contrast with some specific use cases. The diversity of limited-output tES used cases (for example either enhancing alertness and meditation; [50–52]) is precisely why warnings/precautions regarding use cases should be developed by each manufacturer following the risks management process described in LOTES-2017. For example, in relation to driving, tDCS has been explored to enhance driving [53,54], while other forms of limited output tES may be distracting (e.g., phosphenes from tACS; [55, 56]) or specifically intended to produce drowsiness [17,57].

For similar reasons related to both ambiguity and contrast with existing use-cases, blanket precautions against use when “intoxicated” or “incapacitated” are not useful. For example, limited-output tES is extensively trialed for reduced craving against addiction [58–61]. Moreover, many applications of limited-output tES, notably its use in pain control, are intended to reduce reliance on drugs. Limited-output tES is also tested to treat individuals who may be incapacitated such as patients who are minimally-conscious or under ventilation [62–66].

Warning against stimulation “across the chest” may be ambiguous including in light of extensive approaches using extracephalic montages [67,68] including where cardio-respiratory and autonomic functions were monitored [67,69]. Notwithstanding a 1964 case report for transient “respiratory arrest”, we are aware of no modern consensus statement on limited output tES safety that indicate current across the brainstem or chest, as would be produced by an extracephalic montage, is hazardous [23,33]. Moreover, some approaches outside of the LOTES scope such as transcutaneous spinal cord stimulation intentionally apply current across the chest [70,71].

The context for additional changes regarding use in perinatal care and children are discussed separately in later sections.

#### 5.1.1. Warnings (LOTES-2023 update)

For clinical and wellness use we recommend that the user manual provide the following warnings. Warnings may be expanded by relevant additional warnings based on product risk management or as imposed by regulatory agencies, or conversely may be abbreviated under justified rationale so as to not conflict with the intended use (e.g., a device intended for skin wound healing):

- If you are under the active care of a physician, consult with your physician before using this device.
- Do not use without consulting your physician if you have a preexisting neurological or neuropsychiatric condition.
- Do not use in treatment of children, except under medical oversight.
- Apply stimulation electrodes to locations only as directed*.
- Apply stimulation only to intact, clean, healthy skin.
- Do not apply stimulation over open wounds or rashes, or swollen, red, irritated, infected, or inflamed areas or skin eruptions.
- As relevant, follow any instructions on the preparation of the skin including concerns about hair products, make up, and other topical skin products.
- Do not use when the user and/or device is likely to get wet (outside of any supplied sponges as directed).
- Unsupervised use in children is not recommended.
- Do not use this device while intoxicated or incapacitated**.
- If you are pregnant or may be pregnant, consult your perinatal health team prior to using this device.
  * As relevant to device use, may direct to avoid applying stimulation across the chest.
  * * Would not apply for some devices such as those specifically intended for use during sleep or to manage addiction.

#### 5.1.2. Precaution (LOTES-2023 update)

We recommend that the user manual also should afford the following precautions. These precautions may be expanded by relevant additional warnings based on product risk management or as imposed by regulatory agencies, or conversely may be abbreviated under justified rationale (e.g., a device which by design increases intensity so sensation during treatment course):

- You may experience (mild and transient) skin irritation (erythema) or hypersensitivity due to the electrical stimulation or skin contact medium (gel or saline) - this should resolve after stimulation (*<*24 h). Avoid re-applying stimulation if skin changes are not resolved before next session.
- If you have a chronic skin disorder, such as psoriasis or otherwise sensitive skin, pay close attention to and stop if you experience skin irritation.
- Skin sensations from stimulation should remain relatively consistent or decrease during stimulation, if you experience significantly increasing levels of discomfort, stop the stimulation*.
- Use caution if stimulation is applied over areas of skin that lack normal sensations.
- Keep this device out of the reach of unsupervised children.
- Use this device only with the leads, electrodes, and accessories recommended by the manufacturer.

### 5.2. Pediatric LOTES-2023 update

In the LOTES-2023 update, we update the LOTES-2017 phrasing of precaution regarding use in pediatric populations in light of new data made available in the past five years. Recent reviews of pediatric tDCS now include data from *>*40 studies, treating hundreds of children between the age of 6–17 with thousands of tES sessions [72,73]. Based on this now much larger pediatric tDCS data set, no indication for any pediatric-specific side effects of tDCS were reported. Similar to adult tDCS applications [9], also in pediatric tDCS, no adverse events have hitherto occurred, in line with the overall tES literature consistently documenting tDCS as a well-tolerated neuromodulation technique [73].

In clinical trials, tDCS has now been investigated in pediatric populations for *>*10 years with no reported increase in side effects or concerns about tolerability [74]. Pediatric tDCS tends to be mainly investigated as a therapeutic intervention in CP, ADHD, Autism spectrum disorder, and learning disorders. Additional studies of tDCS in pediatric applications include tourette’s/tic disorder, schizophrenia and eating/alcohol disorders [72].

In terms of stimulation protocol and parameters, pediatric tDCS is often limited to ≤ 2 mA, with session durations often reduced to 20 min. However, those pediatric studies that did use standard 2 mA and 30-min session durations also reported no increase in side effects (See for example [75,76]). Thus, tDCS doses common in adult populations (e.g. 2 mA) are considered safe in pediatrics [73], those outcomes (dose response) may vary [77]. Overall tDCS treatment duration in pediatric populations has so far not exceeded 20 consecutive days of stimulation [78,79]. Hence, whereas a limited number of adult tDCS studies have reported overall tDCS treatment durations of up to 6 months of daily stimulation without side effects [80], such longer term use data has not yet been explored in pediatric tDCS applications.

In 2019, the FDA permitted marketing of external Trigeminal Nerve Stimulation (eTNS) for attention deficit hyperactivity disorder (ADHD) in children 7–12 years old. The device is used at home under supervision of a caregiver, under prescription for use during sleep for four weeks. The FDA indicates “most common side effects observed with eTNS use are: drowsiness, an increase in appetite, trouble sleeping, teeth clenching, headache and fatigue. No serious adverse events were associated with use of the device.” [81].

Current evidence is consistent with tDCS applications in children from 6 years of age without evidence of related major adverse events and transient and self-limiting reports of minor adverse events. In pediatric tDCS, parameters may be adjusted by shortening session duration and lowering intensity relative to adult norms, but this is not a safety requirement. The requirement to protect children must be balanced against the value of allowing clinical trials to study treatments. Thus, standards and labeling must be evidence-based. Relatedly, the FDA has now established the Pediatric Research Equity Act [82] to include children in care as relates to drugs if the children are affected by the target condition.

Recommendations for baseline (i.e., applied to every device) warnings cannot rationally exclude accepted indications for use (e.g., FDA approval of eTNS for children during sleep).

### 5.3. Limited-output tES during pregnancy

In the LOTES-2017 the use of tDCS in pregnant women is recommended as follows:

WARNING: *“If you are pregnant or may be pregnant, you should follow precautions recommended by your physician.”*

PRECAUTION: “The safety of electrical stimulation during pregnancy has not been established”.

These warning and precaution regarding pregnant women were stated based on the lack of systematic data regarding safety outcomes when using limited-output tES during pregnancy. Therefore, we aim to update these recommendations based on systematic reviews of the literature currently available about the use of non-invasive brain stimulation to manage depressive symptoms during pregnancy.

Pacheco et al. [83] found five reports about the use of tES in pregnancy. Of these, tDCS use was reported in one conference abstract presenting the preliminary results of an open-label single-arm pilot study with three patients [84], one case report [85], and one small RCT with 10 patients in the active group [86]. Trigeminal Nerve Stimulation use was reported in one case study [87], and tACS was reported in another case study [88].

On the March 12, 2022, we repeated the search in PubMed, adding “tPCS” to the search terms. One new published report was found [89] which is an extension of Palm et al. (2017) [84]. Table 2 depicts the characteristics of the studies included and in Table 3 we present a summary of the reports regarding safety across the included studies.

In what concerns tDCS, we found three studies reporting its use to manage depressive symptoms during pregnancy (we consider Palm et al., 2017 [84] and Kurzeck et al., 2021 [89] as one single study), that included a total of 16 women between the first and second trimester of pregnancy. Overall, 330 tDCS sessions were completed.

The primary diagnosis across these three tDCS studies was MDD with all patients being medication-free and starting stimulation while pregnant but varying across trimesters. tDCS parameters were the same in all studies: the stimulation site was the DLPFC, with the anode placed over the F3 and the cathode over the F4, using a current intensity of 2 mA. Stimulation was applied once or twice daily during 10–30 sessions.

Women reported to have experienced mild fleeting phosphenes and transient common side effects expected from tDCS (headache during and right after the stimulations, and mild burning sensations and itching at application site). No severe adverse outcomes were observed both for mothers or the fetus during pregnancy.

Follow-up up to delivery and postpartum was not performed across studies, except for Vigod et al. (2019) [86] that reported one preterm birth occurring in the tDCS group but no direct relation with tDCS was established. Kurzeck et al. (2021) [89] assessed two patients after delivery and reported no side effects or negative outcomes for the new mothers or the newborns.

A recent consensus review [23] on ‘Low intensity transcranial electric stimulation: Safety, ethical, legal regulatory and application guidelines’ concluded:

“The profile of AEs in terms of frequency, magnitude and type is comparable in healthy and clinical populations, and this is also the case for more vulnerable populations, such as children, elderly persons, or pregnant women …, although risks for the embryo or fetus during TES are logically negligible, the risk is actually unknown, and it should be recognized that any research on medical products in pregnant women is regulated by law.”

Notwithstanding arguments based on ‘appeal to ignorance’ and that emerging human trials are consistent with no known risk to the fetus, the direct consideration of the amount of electrical current that may flow from electrodes on the head to the fetus suggest non-active intensities. Specifically, computational models of a pregnant female have predicted the intensity of electric field around the fetus during transcranial stimulation. These simulated were for Electroconvulsive-Therapy (ECT) but can be scaled down (from ~1 A to ~1 mA) for limited-output tES. The electric fields produced around a fetus are 0.02% of the electric field in the parents’ brain [90]. For limited-output tES this correspond to ~0.00005 V/m. Such low electric fields are not known to be physio-logical active (much less hazardous), moreover so for the limited time tES is applied. Technologies which produce orders of magnitude higher electric fields in the body, namely TMS and ECT, are trialed in pregnant individuals. For example, the updated Rossi et al. guidelines [6] indicated: “a cautious conclusion can be made that rTMS is minimal risk for the mother and child.

Notwithstanding the required legal special protections for pregnant individuals (including in human trials) and the increased vigilance generally warranted, based on current data, the safety profile and high tolerability of limited-output tES seen in other adult populations seems to be confirmed during pregnancy as far as there is no concomitant pharmacological treatment and stimulation parameters are within those tested so far. Arguments for restriction (or prohibition) based on (the logical fallacy) of appeal to ignorance (*ad ignorantiam*) have a cost when the use-cases for limited output-tES in pregnancy are considered (namely potentially lifesaving for both parent and fetus).

#### 5.3.1. Updated LOTES-2023 guidelines on use in pregnancy

Based on existing evidence there is no indication that limited-output tES is unsafe to use during pregnancy both for mothers and the fetus within the described stimulation parameters, duration of sessions and duration of treatment. Treatment alternatives should be discussed with a perinatal mental health physician. We note, untreated perinatal mood and anxiety disorders are associated with negative obstetric and neurodevelopmental outcomes [91].

Available scientific evidence does not support the need of a general ‘Precaution’ against the use of limited-output tES during pregnancy. Therefore, we recommend removing limited-output tES applications during pregnancy from the general ‘Precautions’ in LOTES-2023. Two ‘Warnings’, one general (“If you are under the active care of a physician …”) and one specific (“If you are pregnant or may be pregnant …”) remain. These changes are neither a prohibition nor an endorsement for use of limited-output tES in pregnancy for all or any given indication.

### 5.4. Limited-output tES (tDCS) and breastfeeding

The use of limited-output tES by postpartum women including those currently breastfeeding does not require special warnings (or special consideration beyond typical adults). Contrary to pharmacological treatments, there is no rationale for an impact on breastmilk.

For example, due to its safe profile and clinical efficacy, tDCS is a promising alternative treatment for women diagnosed with postpartum depression and currently breastfeeding. Ongoing and future clinical trials aimed at testing tDCS antidepressant efficacy in postpartum breastfeeding women will lead towards having tDCS as a choice of treatment within the risk/benefit calculation, among those currently recommended by the Clinical Practice Guidelines.

## Figures and Tables

**Table 1:** Age distribution.

Age		Number of users						Number of stimulations
		Total		Male		Female			

6–9		141 (2.9%)		112 (2.3%)		29 (0.6%)		5548 (3.2%)
10–19		786 (16.2%)		477 (9.8%)		309 (6.4%)		21,562 (12.5%)
20–29		1267 (26.0%)		599 (12.3%)		668 (13.7%)		68,591 (39.8%)
30–39		735 (15.1%)		327 (6.7%)		408 (8.4%)		18,850 (10.9%)
40–49		527 (10.8%)		226 (4.6%)		301 (6.2%)		13,925 (8.1%)
50–59		433 (8.9%)		146 (3.0%)		287 (5.9%)		10,405 (6.0%)
60–69		377 (7.7%)		119 (2.4%)		258 (5.3%)		11,527 (6.7%)
70–79		388 (8.0%)		120 (2.5%)		268 (5.5%)		12,337 (7.2%)
80–89		204 (4.2%)		67 (1.4%)		137 (2.8%)		9597 (5.6%)
90+		8 (0.2%)		1 (0.0%)		7 (0.1%)		194 (0.1%)
Total		4866 (100.0%)		2194 (45.1%)		2672 (54.9%)		172,536 (100.0%)

- Subgroup analysis by site								

Age	At clinic use				At home use			
	# of user	# of stimulations	# of stimulation per user (times/person)		# of user	# of stimulations	# of stimulation per user (times/person)	

6–9	114 (3.6%)	1535 (4.0%)	13.5		55 (2.9%)	4013 (3.0%)	73.0	
10 –19	625 (19.8%)	10,664 (28.1%)	17.1		218 (11.3%)	10,898 (8.1%)	50.0	
20–29	870 (27.6%)	8442 (22.3%)	9.7		450 (23.4%)	60,150 (44.7%)	133.7	
30–39	497 (15.7%)	4922 (13.0%)	9.9		257 (13.4%)	13,927 (10.3%)	54.2	
40–49	344 (10.9%)	3182 (8.4%)	9.3		203 (10.6%)	10,743 (8.0%)	52.9	
50–59	270 (8.6%)	2839 (7.5%)	10.5		188 (9.8%)	7567 (5.6%)	40.3	
60–69	210 (6.7%)	2849 (7.5%)	13.6		172 (9.0%)	8679 (6.4%)	50.5	
70–79	155 (4.9%)	2383 (6.3%)	15.4		236 (12.3%)	9952 (7.4%)	42.2	
80–89	69 (2.2%)	1081 (2.9%)	15.7		136 (7.1%)	8518 (6.3%)	62.6	
90+	3 (0.1%)	21 (0.1%)	7.0		6 (0.3%)	201 (0.1%)	33.5	
Total	3157 (100.0%)	37,918 (100.0%)	12.0		1921 (100.0%)	134,648 (100.0%)	70.1	

**Table 2:** Characteristics of the included studies.

Study	Study Design	# Participants	Concomitant treatment	Trimester at start of TES	Type of tES	Anode	Cathode	Current Intensity	Electrodes/ Sponges’ dimensions	Duration of Stimulation	# Sessions

Trevizol et al., 2015	case report	1	No	second to third	tACS	supraorbital trigeminal branches (V1) bilaterally		120 Hz	20 cm^2^	30 min	10
Sreeraj et al., 2016	case report	1	No	first, second. and third	tDCS	F3	F4	2 mA	25 cm^2^	30 min	10
Palm et al., 2017	single arm	3	No	first, second and third	tDCS	F3	F4	2 mA	35 cm^2^	30 min	30
Vigod et al., 2019	RCT	20 10 active +10 sham	No	second to third	tDCS	F3	F4	2 mA	35 cm^2^	30 min	15
Wilkening et al., 2019	case report	1	No	first	TNS	F3	F4	40 Hz at 2 mA	35 cm^2^	20 min	9
Kurzeck et al., 2021	single arm	6	No	first, second and third	tDCS	F3	F4	2 mA	35 cm^2^	30 min	30

**Table 3:** Safety reports of the included studies.

Study	Adverse effects (mothers)	Neonatal safety

Trevizol et al., 2015	Not reported	Not observed
Sreeraj et al., 2016	Transient, mild burning sensations at application site and transitory experience of phosphenes	Not reported
Palm et al., 2017	Not reported	Not reported
Vigod et al., 2019	Not reported	Not observed
Wilkening et al., 2019	Mild phosphenes during stimulation	Not reported
Kurzeck et al., 2021	Mild headache during and right after the stimulations; itching sensation beneath the electrodes; insomnia (probably nonrelated to tDCS); phosphenes (3 patients). No severe effects	No irregularities of fetal health detected on regular obstetric observations, including heart rate measurement. No severe adverse effects
